# Trends and associated factors in the uptake of HIV testing among female sex workers in Sino-Vietnam border areas in Guangxi, China: a cross-sectional study

**DOI:** 10.1186/s12879-022-07459-3

**Published:** 2022-05-19

**Authors:** Bingyu Liang, Qianni Huang, Yanyun Ou, Fei Zhang, Peidong Zhang, Aidan Nong, Shide Mo, Zhenxian Wu, Hai Xie, Huayue Liang, Jie Liu, Junjun Jiang, Hao Liang, Deping Liu, Li Ye

**Affiliations:** 1 Guangxi Key Laboratory of AIDS Prevention and Treatment, School of Public Health, Guangxi Medical University, Nanning, 530021 Guangxi China; 2 Guangxi Collaborative Innovation Center for Biomedicine, Life Sciences Institute, Guangxi Medical University, Nanning, 530021 Guangxi China; 3 Chongzuo Center for Disease Control and Prevention, Chongzuo, 532200 Guangxi China; 4 Fangchenggang Center for Disease Control and Prevention, Fangchenggang, 538000 Guangxi China

**Keywords:** HIV, AIDS, Female sex worker, Sino-Vietnam border, HIV testing, Migrant

## Abstract

**Background:**

HIV testing is a primary prevention strategy against the HIV epidemic and an entry point for HIV/AIDS-related care, prevention and treatment. This study aimed to estimate the uptake of HIV testing among Sino-Vietnam female sex workers (FSWs) in Guangxi, China, from 2016 to 2018, and to identify the factors influencing HIV testing uptake.

**Methods:**

A cross-sectional survey was conducted among FSWs in two Sino-Vietnam border cities of Guangxi, China. The survey was conducted through face-to-face interview, the data were collected by a structured questionnaire, and HIV-1 infection was detected simultaneously. Logistic regression analysis was used to determine the factors associated with uptake of HIV testing.

**Results:**

In total, 4565 Chinese local FSWs (CL-FSWs) and 636 cross-border migrant Vietnamese FSWs (CMV-FSWs) were recruited into this study. HIV-1 prevalence in CL-FSWs and CMV-FSWs was 0.70% and 3.14%, respectively. The rate of self-reported uptake of HIV testing in CL-FSWs and CMV-FSWs was 54.56% and 45.44%, respectively. The rates of self-reported uptake of HIV testing declined in both groups from 2016 to 2018. Logistic regression analysis indicated that a number of factors, including socio-demographic characteristics (age < 35 years, higher education, location in Chongzuo City), behaviour factors (having received free AIDS education, condom distribution services and peer education services, high risk sexual behaviours such as inconsistent condom use, having regular commercial sexual partners, etc.), psychological factors (perception of vulnerability to HIV/STIs, perception of risk for HIV infection) were the factors significantly related to uptake of HIV testing.

**Conclusion:**

In recent years, the rate of HIV testing uptake among Sino-Vietnam border FSWs in Guangxi, China was low, which may be an important reason for the high HIV-1 prevalence among FSWs in the region. A number of factors were identified to be associated with HIV-1 testing uptake, suggesting that HIV testing prevention strategies in this region could include accelerating AIDS education training, raising personal awareness of HIV testing, and improving accessibility of HIV testing.

**Supplementary Information:**

The online version contains supplementary material available at 10.1186/s12879-022-07459-3.

## Background

HIV testing, a key policy response to the HIV/AIDS epidemic, has been promoted as a primary prevention strategy and an entry point for assessing HIV/AIDS-related services, such as treatments and the subsequent improvements in life expectancy. Expanding HIV testing not only increases the proportion of HIV-positive people who know their infection status, but also provides an effective method of secondary prevention for those who test positive. China's HIV testing system has been developed over a period of time and has gone through several stages, including three testing models: voluntary counselling and testing (VCT), high-risk group testing, and provider-initiated testing and counselling (PITC) [1]. However, a recent survey showed that there was still a large gap between high willingness rate and low uptake rate of HIV testing in China during 2017–2018 [2]. The National Health Commission of the People’s Republic of China estimated that by the end of 2018, only two-thirds of people living with HIV knew their HIV status [3], which was far from the aim of 95% of all HIV-infected individuals being diagnosed by 2030 proposed by the Joint United Nations Programme on HIV/AIDS (UNAIDS) [4]. Female sex workers (FSWs), a high-risk population for HIV infection, are a major bridge for HIV transmission that may determine the speed of HIV transmitting from high-risk groups to the general population. A systematic review in 2012 noted that the risk of HIV infection among FSWs is 50 times higher than that of the general female population in low-income and middle-income countries [5]. Cross-border migrant FSWs are of particular concern because of the relatively higher HIV prevalence in this population [6–8]. The HIV testing rate was low among cross-border FSWs due to language barriers, the invisibility of sex work, and the limited availability of HIV prevention services [8]. Previous studies have shown that nearly half of FSWs in China had not been tested for HIV in the past year [9, 10]. The situation is even worse in Guangxi, where only 35.9% of FSWs have been tested for HIV during 2012–2015 [11], greatly limiting the access to HIV treatment and increasing the likelihood of HIV transmission to the general population.

Guangxi, a province in the Southwest of China that borders Vietnam and the ‘Golden Triangle’, is a gateway for drug trafficking routes and cross-border migration of sex workers. By the end of 2017, Guangxi had the second highest number of HIV reported cases among provincial-level regions in China, accounting for 12% of the total national HIV reported cases, and heterosexual intercourse had become the primary transmission route in Guangxi [12]. The northern Vietnam region bordering Guangxi is a mountainous area, where the economy is relatively underdeveloped. Many Vietnamese women went to Guangxi to find temporary work [13]. A review in 2020 summarized 51 publications and indicated that the HIV prevalence among FSWs in Guangxi ranged from 0.13% to 6.78% during 2008–2018, higher than those of other areas in China [14]. Our previous study found that the proportion of newly diagnosed HIV patients with late HIV presentation was very high in Guangxi [15]. Lack of awareness and timely HIV testing among high-risk populations leads to high rates of late HIV presentation, advanced HIV disease, poor treatment effectiveness and high mortality [15]. Therefore, strengthening HIV testing among high-risk groups and reducing HIV transmission through sexual behavior have become an urgent problem to be solved in Guangxi.

A number of studies have documented many factors associated with uptake of HIV testing among FSWs in China, including older age [16], less education [16], working in higher-income venues [16], better HIV knowledge [16], financial and time costs [17], stigmatization and discrimination [17], higher perceived risk, condom use, a longer history of sex work, higher VCT knowledge [18], etc. Although there are many publications on HIV testing and related factors in China, data from Guangxi province, which has a high HIV disease burden, is quite limited, especially from Sino-Vietnam border areas. The purpose of this study was to investigate the uptake of HIV testing among Sino-Vietnam border FSWs in Guangxi, China, and to explore the relevant influencing factors.

## Methods

### Study setting

From 2016 to 2018, a cross-sectional survey was conducted among FSWs in two Sino-Vietnam border cities (Chongzuo City with seven counties and one district, Fangchenggang City with two counties and two districts) from May to July every year. The two cities share a 637-km border with northern Vietnam and have eleven land ports [19].

### Study participants and procedures

The participants were recruited from a variety of venues where the local Center for Disease Control and Prevention (CDC) provided intervention services for HIV high-risk populations. Random sampling was conducted on venues, and cluster sampling was performed on FSWs of the selected sites. The venues included high- or middle (mid)—tier venues and low-tier venues based on the mean income of FSW per transaction [11, 20] and the condition of the venue [21]. Due to the underdeveloped economy in the Sino-Vietnam border areas in Guangxi, the high-tier venues only accounted for a small percentage (3.33%) in our study. Therefore, our study grouped high- and mid- tier venues into one group (high- or mid- tier venues), including sauna or bathing centres, nightclubs, karaoke bars, dance halls, pub bars, upscale or mid-range hotels. Low tier venues included hair salons, foot bath centers, small restaurants, rental houses, parks and streets. The inclusion criteria of FSWs for this study were as follows: (1) females aged 16 years and over; (2) self-reported charging for sex transaction more than four times per month in the past 6 months; (3) self-reported HIV negative or unknown HIV status; (4) able to understand the contents of the questionnaires and provide verbal and written consent. Detailed descriptions of sampling and recruitment can be found in our previous study [11].

After obtaining informed consent from the participants, trained CDC staff conducted face-to-face interviews in a private room, using a structured questionnaire. During the survey, interviewers provided explanations and instructions to help participants understand any questions in the questionnaire. For migrants with limited Chinese language proficiency, a Vietnamese-Chinese interpreter assisted in the survey. After completing the questionnaire, each participant was compensated 50 RMB (about $7 USD) for the participation. This study was approved by the Human Research Ethics Committee of Guangxi Medical University (ethical review No. 2013-130).

### Socio-demographic, behavioural, HIV/AIDS-related knowledge, and psychological variables

The survey collected socio-demographic data including nationality, age, ethnicity, marital status, education, and current location. According to nationality information, the participants were divided into two groups: Chinese local FSWs (CL-FSWs) and cross-border migrant Vietnamese FSWs (CMV-FSWs).

Behavioural variables were measured by questions regarding condom use, commercial sex transaction, injected illicit drug, their regular commercial or non-commercial sexual partners, STI history, having received HIV-related services, self-reported uptake of HIV testing, etc.

HIV/AIDS-related knowledge was assessed with an 8-question evaluation form proposed by the Chinese CDC [22], which is widely used in China [23, 24]. Participants who correctly answered six or more questions were considered to have adequate HIV/AIDS-related knowledge, and those who correctly answered five or less questions were considered to have low HIV/AIDS-related knowledge [22].

Psychological variables mainly referred to the perceived risk of HIV infection and the perception of vulnerability to HIV/STIs. The assessment of the perceived risk for HIV infection was scored using four-option questions. The assessment of perception of vulnerability to HIV/STI was scored using Yes/No questions, and the two-item scale had a Cronbach’s alpha value of 0.80. In addition, we investigated the participants’ perceptions of family attitudes toward their work.

The behavioural variables, assessment of HIV/AIDS-related knowledge and psychological variables can be seen in Additional file 1 ‘Key population quantitative questionnaires’.

### HIV testing

At the time of the survey, participants were asked to provide blood specimens to test for HIV antibodies. Specimens that screened positive by enzyme-linked immunosorbent assay (ELISA) (Wantai Biological Pharmaceutical Co., Beijing, China) were confirmed by western blot (WB) assay (HIV Blot 2.2 WB; Genelabs Diagnostic, Singapore). The purpose of the HIV testing is to estimate the HIV prevalence among FSW population.

In addition, the questionnaire presented the option of self-reported uptake of HIV testing in the preceding year. “HIV Testing” in the questionnaire refers to the history of HIV testing, which is self-reported, using either ELISA or WB.

### Statistical analysis

The collected data were double entered with EpiData3.0. For socio-demographic characteristics, the proportions were calculated for the two groups (CL-FSWs and CMV-FSWs). The chi-square trend test was used to test the significance of time trend. Univariate logistic regression analyses were performed for each independent variable of uptake of HIV testing, with crude odds ratio and 95% confidence intervals (CI) indicated. Variables with significant univariate level (p < 0.1) were input into the logistic regression model to calculate the adjusted ORs and 95% CIs against the dependent variable for each group. Finally, a multivariate logistic regression model was established by the stepwise method. Statistical significance was defined as p < 0.05 (two-tailed test). All analyses were performed using SPSS statistical package version 23.

## Results

### Socio-demographic characteristics of FSWs

As shown in Table 1, a total of 5201 FSWs who self-reported HIV-negative or unknown status were recruited. Of them, 2781 (53.47%) had been tested for HIV in the preceding year. The median age of the participants was 33.9 years (25.4–42.4 years). The majority of the participants were Chinese (87.77%), of Zhuang and other ethnicities (65.85%), married or cohabiting (67.76%), educated for 9 years or more (62.51%) and living in Chongzuo City (70.12%).

**Table 1:** Socio-demographic characteristics of FSWs in Sino-Vietnam border, China (n = 5201)

Variables	Total (n = 5201) n, (%)	Cross-border migrant Vietnamese FSWs (n = 636) n, %		Chinese local FSWs (n = 4565) n, %	
		n	%	n	%
Tested for HIV in the preceding year					
Yes	2781 (53.47)	289	45.44%	2492	54.59%
No	2420 (46.52)	347	54.56%	2073	45.41%
Age					
≥ 35	2905 (55.85)	413	64.94%	2492	54.59%
< 35	2296 (44.15)	223	35.06%	2073	45.41%
Ethnicity					
Han	1776 (34.15)	–	–	1776	38.90%
Zhuang	2396 (46.07)	2	0.31%	2394	52.44%
Other	1029 (19.78)	634	99.69%	395	8.65%
Marital status					
Married or cohabiting	3524 (67.76)	431	67.77%	3093	67.75%
Single or divorced	1677 (32.24)	205	32.23%	1472	32.25%
Education					
< 9 years	1950 (37.49)	267	41.98%	1683	36.87%
≥ 9 years	3251 (62.51)	369	58.02%	2882	63.13%
Current location					
Chongzuo	3647 (70.12)	386	60.69%	3261	71.43%
Fangchenggang	1554 (29.88)	250	39.31%	1304	28.57%

### HIV prevalence, HIV testing and receiving HIV-related prevention services among FSWs

**Fig. 1:**
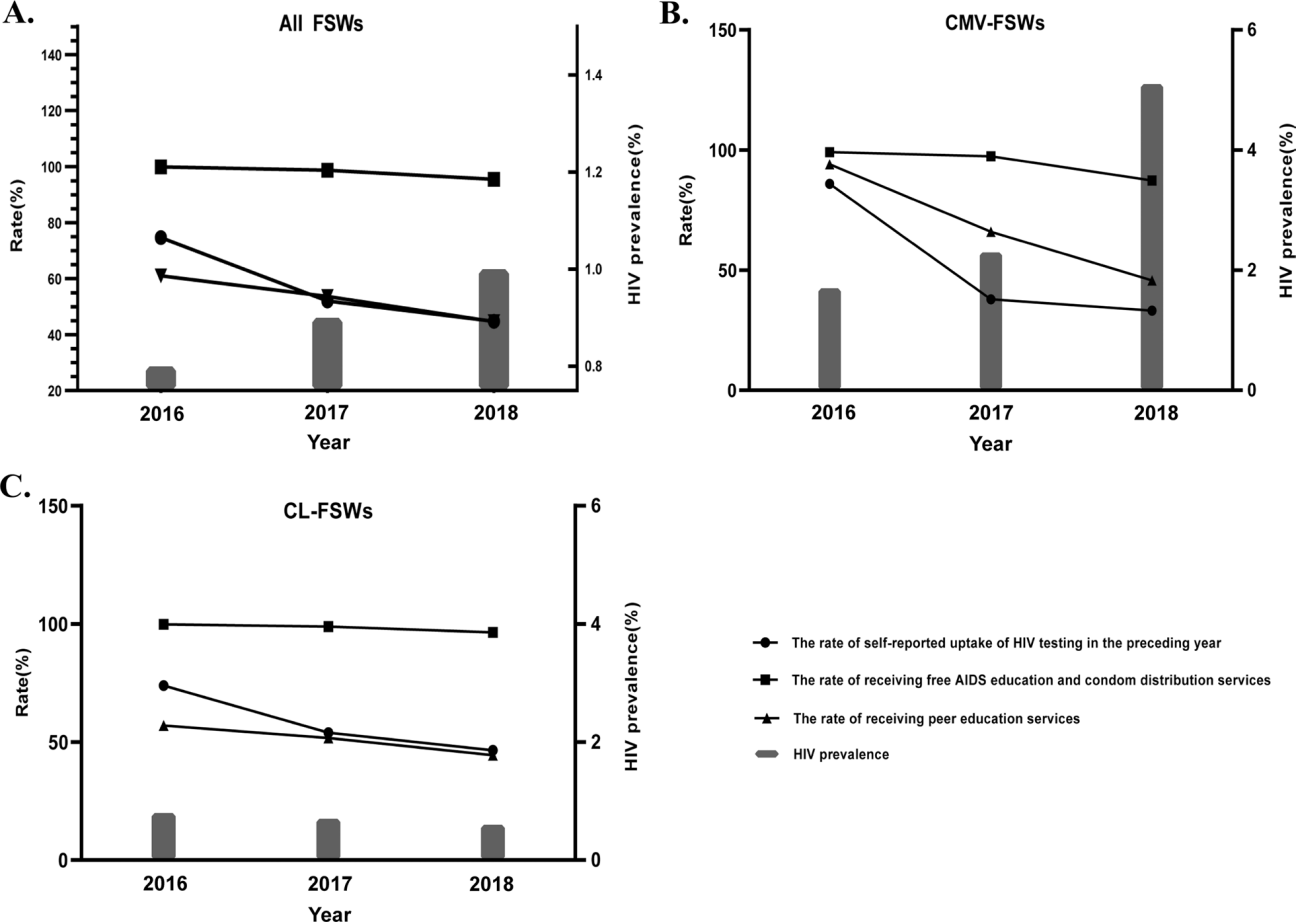
Trends in HIV testing, HIV prevalence and prevention services. **A** Trends in HIV testing, HIV prevalence and receiving HIV prevention services among all FSWs in the Sino-Vietnam border region, 2016–2018. **B** Trends in HIV testing, HIV prevalence and receiving HIV prevention services among CMV-FSWs in Sino-Vietnam border region, 2016–2018. **C** Trends in HIV testing, HIV prevalence and receiving HIV prevention services among CL-FSWs in Sino-Vietnam border region, 2016–2018

The HIV prevalence among all FSWs was 0.77% (2016), 0.91% (2017), and 1.12% (2018) (Fig. 1A). Compared with CL-FSWs, CMV-FSWs had a significant higher HIV prevalence (3.12%. vs. 0.70%) (Fig. 1B, C). The rate of self-reported uptake of HIV testing among all FSWs, CL-FSWs, and CMV-FSWs was 53.47%, 54.6% and 45.4%, respectively (Fig. 1A–C). As shown in Fig. 1A, the rate of self-reported uptake of HIV testing decreased significantly from 2016 to 2018 (linear trend χ^2^ = 187.04, p < 0.001). Similarly, the rate of receiving free AIDS education and condom distribution services, peer education services were also significantly decreased. In the CMV-FSWs group, the HIV prevalence increased significantly (linear trend χ^2^ = 3.73, p = 0.05), but the rates of self-reported uptake of HIV testing in the preceding year, receiving free AIDS education and condom distribution services, and peer education services declined significantly (Fig. 1B). In the CL-FSWs group, the HIV prevalence did not change significantly (linear trend χ^2^ = 0.25, p = 0.62), but the rates of self-reported uptake of HIV testing in the preceding year, receiving free AIDS education and condom distribution services, and peer education services declined significantly (Fig. 1C).

### Univariate analyses of factors associated with uptake of HIV testing in the preceding year

Univariate analysis indicated that, for socio-demographic variables, in both groups, uptake of HIV testing in the preceding year was significantly related to age < 35 years, married or cohabiting status, 9 years' education or longer, location in Chongzuo City (Table 2). For behaviour variables, in both groups, HIV testing was significantly related to working in high-/mid-tier of venue, having stayed for more than 6 months in the current residence, inconsistent condom use in the previous month, history of self-reported STIs, etc. (Table 3). For psychological variables, perception of vulnerability to HIV/STIs and fear of their families knowing about sex work were the factors associated with HIV testing (Table 3).

**Table 2:** Univariate and multivariate logistic regression of socio-demographic factors for HIV testing in the preceding year among Sino-Vietnam border female sex workers, 2016–2018 (n = 5201)

Variables	Total tested	Cross-border migrant Vietnamese FSWs (n = 636)					Chinese local FSWs				
		Tested	COR^▼^ (95% CI)	*p*	AOR^▼^ (95% CI)	*p*	Tested	COR^▼^ (95% CI)	*p*	AOR^▼^ (95% CI)	*p*
Age											
≥ 35 years old	1230 (44.23)	164 (39.71)	1		1		1066 (42.78)	1		1	
< 35 years old	1551 (55.77)	125 (56.05)	1.94 (1.39–2.69)	< 0.001	1.93 (1.20–3.10)	0.01	1426 (68.79)	2.95 (2.61–3.33)	< 0.001	1.89 (1.60–2.24)	< 0.001
Ethnicity											
Han	958 (34.45)	–	–	–	–	–	958 (53.94)	0.85 (0.68–1.06)	0.15	–	–
Zhuang	1307 (47.00)	–	–	–	–	–	1305 (54.51)	0.87 (0.70–1.08)	0.20	–	–
Other	516 (18.55)	–	–	–	–	–	229 (57.97)	1			
Marital status											
Married, cohabiting	2069 (74.40)	217 (50.35)	1.87 (1.33–2.64)	< 0.001	1.34 (0.84–2.13)	0.22	1852 (59.88)	1.94 (1.71–2.20)	< 0.001	0.94 (0.80–1.11)	0.46
Single	712 (25.60)	72 (35.12)	1		1		640 (43.48)	1		1	
Education											
≥ 9 years	1705 (61.30)	209 (78.28)	3.05 (2.19–4.26)	< 0.001	1.75 (1.09–2.82)	0.02	1496 (88.89)	0.74 (0.66–0.84)	< 0.001	1.13 (0.96–1.33)	0.13
< 9 years	1076 (38.70)	80 (21.68)	1		1		996 (34.56)	1		1	
Location											
Chongzuo	2358 (84.79)	238 (61.66)	6.28 (4.34–9.08)	< 0.001	3.23 (1.70–6.11)	< 0.001	2120 (65.01)	4.66 (4.05–5.36)	< 0.001	3.67 (3.07–4.38)	< 0.001
Fangchenggang	423 (15.21)	59 (0.24)	1		1		372 (28.53)	1		1	

*COR* crude odds ratio, *AOR* adjusted odds ratio, *CI* confidence interval

**Table 3:** Univariate and multivariate logistic regression of behavioral factors and psychological factors for HIV testing in the preceding year among Sino-Vietnam border female sex workers, 2016–2018 (n = 5201)

		Cross-border migrant Vietnamese FSWs						Chinese local FSWs			
Variables	Total tested	Tested	COR^▼^ (95% CI)	*p*	AOR^▼^ (95% CI)	*p*	Tested	COR^▼^ (95% CI)	*p*	AOR^▼^ (95% CI)	Tested
Having adequate HIV-/AIDS-related knowledge											
Yes	2681 (96.40)	269 (47.36)	2.16 (1.25–3.73)	0.01	0.85 (0.41–1.80)	0.68	2412 (54.71)	1.15 (0.83–1.58)	0.40	–	–
No	100 (3.60)	20 (29.41)	1		1		80 (51.28)	1		–	–
Working venue type											
High-/Mid-tier	1425 (51.24)	138 (66.99)	3.75 (2.64–5.32)	< 0.001	5.11 (2.83–9.21)	< 0.001	1287 (50.47)	0.68 (0.61–0.77)	< 0.001	1.0 (0.84–1.20)	0.96
Low-tier	1356 (48.76)	151 (35.12)	1		1		1205 (50.80)	1		1	
Age of sexual debut											
< 18 years	442 (15.89)	94 (51.37)	1.40 (0.99–1.97)	0.06	0.69 (0.43–1.09)	0.11	348 (52.97)	0.93 (0.79–1.10)	0.37	–	–
≥ 18 years	2339 (84.11)	195 (43.05)	1		1		2144 (54.86)	1		–	–
Age of commercial sexual debut											
≥ 18 years	2750 (98.89)	277 (45.19)	1		–	–	2473 (55.07)	1		–	–
< 18 years	31 (1.11)	12 (52.17)	1.32 (0.58–3.05)	0.51	–	–	19 (25.68)	0.28 (0.17–0.48)	< 0.001	–	–
Months of staying in the current residence											
≥ 12 months	1626 (58.47)	95 (42.60)	0.66 (0.47–0.93)	0.02	1.1 (0.68–1.83)	0.68	1531 (65.60)	2.06 (1.80–3.67)	< 0.001	1.28 (1.08–1.52)	0.04
6–11 months	322 (11.58)	16 (20.78)	0.23 (0.13–0.42)	< 0.001	0.56 (0.27–1.18)	0.13	306 (35.33)	0.59 (0.50–0.71)	< 0.001	0.58 (0.47–0.71)	< 0.001
< 6 months	830 (29.85)	178 (52.98)	1		1		652 (48.01)	1		1	
Years of FSW experience											
≥ 4 years	1450 (52.14)	79 (43.65)	0.90 (0.64–1.28)	0.57	–	–	1371 (57.92)	1.32 (1.18–1.49)	< 0.001	1.61 (1.38–1.88)	< 0.001
< 4 years	1331 (47.86)	210 (46.15)	1		–	–	1121 (51.00)	1		1	
Consistent condom used in last commercial sex											
Yes	2742 (98.60)	280 (45.45)	1.02 (0.42–2.49)	0.97	–	–	2462 (55.09)	1.61 (0.99–2.62)	0.06	1.55 (0.83–2.87)	0.17
No	38 (1.40)	9 (45.00)	1		–	–	29 (43.28)	1		1	
Consistent condom used in the previous month											
Yes	2540 (91.33)	236 (43.30)	1		1		2304 (54.21)	1		1	
No	241 (8.67)	53 (58.24)	1.83 (1.17–2.86)	0.01	1.94 (1.07–3.53)	0.03	188 (59.68)	1.25 (0.10–1.58)	0.06	1.56 (1.15–2.11)	0.004
History of self-reported STIs											
Yes	154 (5.54)	14 (73.68)	3.48 (1.24–9.79)	0.02	4.25 (1.31–13.76)	0.02	140 (61.14)	1.33 (1.01–1.74)	< 0.001	1.07 (0.79–1.46)	0.79
No	2627 (94.46)	275 (44.57)	1		1		2352 (54.24)	1		1	
Ilicit drug use											
Yes	52 (1.87)	3 (50.00)	1.20 (0.24–6.01)	0.82	–	–	49 (64.47)	1.52 (0.95–2.44)	0.08	–	–
No	2729 (98.13)	286 (45.40)	1		–	–	2443 (54.42)	1		–	–
Serving clients in a fixed venue											
Yes	2514 (90.40)	281 (46.68)	2.84 (1.27–6.39)	0.01	1.08 (0.39–3.04)	0.88	2233 (56.23)	1.66 (1.40–2.00)	< 0.001	1.44 (1.17–1.79)	0.001
No	267 (9.60)	8 (23.53)	1		1		259 (43.60)	1		1	
Having received free AIDS education and condom distribution services in the past year											
Yes	2762 (99.32)	284 (47.33)	5.57 (2.14–14.53)	< 0.001	1.77 (0.58–5.39)	0.32	2478 (55.37)	6.74 (3.80–11.95)	< 0.001	2.93 (1.92–2.67)	< 0.001
No	19 (15.18)	5 (13.89)	1		1		14 (15.56)	1		1	
Having received peer education services in the past year											
Yes	1820 (65.44)	243 (59.27)	5.69 (3.90–8.32)	< 0.001	1.1 (0.59–2.08)	0.76	1577 (69.47)	3.43 ( 3.04–3.88)	< 0.001	2.26 (1.92–2.66)	< 0.001
No	961 (34.56)	46 (20.35)	1		1		915 (39.86)	1		1	
Number of clients in the last month											
< 60	1772 (63.72)	192 (48.73)	1		1		1580 (57.69)	1		1	
≥ 60	1009 (36.28)	97 (40.08)	0.70 (0.51–0.97)	0.03	1.30 (0.85–1.98)	0.24	912 (49.95)	0.73 ( 0.65–0.82)	< 0.001	1.66 (1.41–1.94)	< 0.001
Average age of male clients											
≥ 50 years	820 (29.49)	68 (48.23)	1.16 (0.79–1.68)	0.45	–	–	752 (69.05)	2.23 (1.93–2.57)	< 0.001	1.03 (0.85–1.25)	0.76
< 50 years	1961 (70.51)	221 (44.65)	1		–	–	1740 (50.06)	1		1	
Having regular, non-commercial sexual partners											
Yes	2329 (83.75)	15 (44.12)	0.94 (0.47–1.90)	0.87	–	–	437 (71.75)	2.35 (1.95–2.83)	< 0.001	2.10 (1.63–2.62)	< 0.001
No	452 (16.25)	274 (45.51)	1		–	–	2055 (51.95)	1		1	
Having regular, commercial sexual partners											
Yes	1737 (62.46)	205 (50.74)	1.80 (1.30–2.53)	< 0.001	1.59 (1.03–2.46)	0.04	1532 (60.82)	1.76 (1.56–1.98)	< 0.001	1.46 (1.25–1.70)	< 0.001
No	1044 (37.54)	84 (36.21)	1		1	–	960 (46.92)	1		–	–
Clients' average payment for commercial sex											
< 100 RMB	1174 (42.22)	196 (52.83)	2.07 (1.50–2.87)	< 0.001	2.40 (1.36–3.68)	0.001	978 (59.56)	1.37 (1.21–1.55)	< 0.001	0.81 (0.67–0.97)	0.02
≥ 100 RMB	1607 (57.78)	93 (35.09)	1		1		1514 (51.80)	1		1	
Perception of risk for HIV infection											
High level	363 (13.05)	41 (50.00)	1		1		322 (49.31)	1			
Mid level	2091 (75.19)	238 (47.22)	0.89 (0.56–1.43)	0.64	1.00 (0.58–1.73)	1	1853 (56.67)	1.34 (1.14–1.59)	0.001	–	–
Low level	327 (11.76)	10 (20.00)	0.25 (0.11–0.57)	0.001	0.98 (0.36–2.67)	0.97	317 (49.38)	1.10 (0.80–1.25)	0.98	–	–
Perception of vulnerability to HIV/STIs											
Yes	2220 (79.83)	276 (50.00)	5.46 (2.96–10.10)	< 0.001	2.43 (1.17–5.05)	0.02	1944 (59.56)	2.02 (1.78–2.30)	< 0.001	1.72 (1.45–2.03)	< 0.001
No	561 (20.17)	13 (15.48)	1		1		548 (42.12)	1		1	
Fearing their families knowing about their sex work											
Yes	1880 (67.60)	198 (56.09)	2.69 (1.95–3.74)	< 0.001	0.81 (0.50–1.33)	0.41	1682 (62.25)	2.10 (1.90–2.42)	< 0.001	1.56 (1.34–1.81)	< 0.001
No	901 (32.40)	91 (32.16)	1		1		810 (43.48)	1		1	

*COR* crude odds ratio, *AOR* adjusted odds ratio, *CI* confidence interval

In addition, several factors were relevant with HIV testing for CMV-FSWs or CL-FSWs, respectively. For example, among CL-FSWs, the factors associated with HIV testing included more than 4 years of FSW experience, consistent condom use in last commercial sex, having male clients’ older than 50 years, etc. (Table 3). Besides, a perception of low-level risk for HIV infection was associated with HIV testing among CMV-FSWs, while a perception of middle-level risk for HIV infection was related with HIV testing among CL-FSWs (Table 3).

### Multivariate logistic regression analyses of factors associated with uptake of HIV testing in the preceding year

All variables were tested for multicollinearity, but none of the Pearson’s pairwise coefficients exceeded 0.45, indicating low collinearity of these variables. As shown in Tables 2 and 3, for both groups, age < 35 years, location at Chongzuo City, inconsistent condom use in the previous month, having a regular commercial sexual partner, perception of vulnerability to HIV/STIs were the shared factors significantly related to HIV testing. Being paid on average less than 100 RMB (about 15.1 USA dollars) per client was the positive factor for HIV testing among CMV-FSWs, while it was the negative factor for HIV testing among CL-FSWs.

In addition, several different factors were relevant with HIV testing for CMV-FSWs or CL-FSWs, respectively. Among CMV-FSWs, the factors were completing 9 years of education, working in high- or mid- tier venues, and a history of self-reported STIs (Tables 2, 3). Among CL-FSWs, the factors were having stayed more than 12 months in the current residence, more than 4 years of FSW experience, serving clients in a fixed venue, receiving free AIDS education and condom distribution in the past year, etc. (Tables 2, 3).

## Discussion

This is a large-scale cross-sectional study to investigate the HIV prevalence and uptake of HIV testing among FSWs including cross-border migrant Vietnamese FSWs in the Sino-Vietnam border areas in Guangxi, an HIV high-risk region in south-western China. For the first time, we identified a number of factors associated with uptake of HIV testing among FSWs in this region.

Our study indicated a high HIV prevalence among FSWs in this region (overall HIV prevalence was 0.96%), especially among CMV-FSWs (average HIV prevalence was 3.14%). These data are higher than the general level of FSWs in Guangxi [14]. More importantly, our study indicated that only about half of the FSWs reported a recent HIV testing, which is far below the level recommended by WHO [25, 26] and the ‘95–95–95’ target [4], and may be an important reason for the high HIV prevalence among FSWs in the region.

Despite the Chinese government began free testing service in 2003 and expanded the program to over 10,000 sites by the end of 2018 [27], the actual effectiveness of the service has not yet reached the target of UNAIDS. In our study, the decrease in HIV testing among cross-border migrant FSWs was associated with a decline in access to HIV prevention services, leading to a slight increase in the HIV prevalence that was higher than in the other 28 provinces in China, except Yunnan province [28]. Although the slight decline in HIV testing may be only a short-term phenomenon, local CDCs should take measures to examine the accessibility of HIV prevention services, especially for cross-border migrant FSWs.

Our study indicates that age < 35 years and higher education were significantly related to uptake of HIV testing. The finding of an association between younger age and HIV testing was consistent with previous studies [29–31]. Recent evidence suggests that FSWs living in inconvenient locations have a lower rate of HIV testing [32]. Consistent with this, our findings strengthened the association between location and HIV testing uptake, which may reflect the greater availability of HIV testing services in Chongzuo City.

We also found that HIV testing was significantly associated with a higher rate of risky sexual behaviours such as inconsistent condom use, more years of FSW experience, and serving more male clients. These findings were consistent with previous studies showing that people who engaged in high–risk sexual behaviours were more likely to take HIV tests [31, 33, 34]. This consistency is also reflected in the FSWs’ higher perceived vulnerability to HIV/STIs in this study, which is consistent with a previous study and is a promoter for HIV testing [35]. FSWs who engage in high-risk sex activities may be more concerned about HIV infection and in constant fear, prompting them to take HIV testing to reduce their worries about HIV infection. In this sense, FSWs need to be educated about the actual risks of their work and the reality of HIV infection. In addition, in the present study, having regular sex partners and fear of their families knowing about their sex work were motivating factors for HIV testing, as stable sex partnerships can increase trust, emotional closeness and familiarity [17]. This finding highlights the importance of social support from families, partners and peers in promoting HIV testing.

In our study, HIV testing was associated with a history of self-reported STIs, which has also been reported in previous studies [36]. The reason may be that the Chinese government implemented provider-initiated HIV/syphilis testing and counselling (PITC) services to expand HIV testing for potential HIV-positive individuals in STD clinics. In this regard, STD clinics are undoubtedly convenient places to disseminate materials and videos about HIV/AIDS knowledge. Future strategies could therefore be designed to use STD clinics as educational settings to raise awareness of HIV/AIDS, the perception of vulnerability to HIV/STIs, and the knowledge of the association between HIV and STIs. In addition, our study shows that FSWs with perception of vulnerability to HIV/STIs appear to be more likely to have undergone HIV testing, similar to observations in other studies [37, 38], suggesting that inadequate awareness of the high risk of HIV is the barrier to uptake HIV testing. It is therefore important to raise the awareness of FSWs’ perceived risk of HIV infection by reports on the seriousness of the local HIV epidemic through television, newspapers, or new media tools such as Wechat or Tiktok.

It is worth mentioning that whether CL-FSWs had received free AIDS education and condoms, and whether they had received peer education from HIV prevention programs were the two most important factors influencing uptake of HIV testing, which is consistent with previous studies [18, 37, 39]. It has been reported that exposure to community services, especially when tested individuals talked openly about HIV, can lead to a greater acceptance and uptake of testing, possibly due to peer influence [40]. Access to specific HIV prevention programmes is also a key factor in promoting HIV testing. These findings highlight the importance of peer relationships for HIV education and promotion of HIV testing services in the context of social discrimination against participants who often hide their sex work from their families. Meanwhile, the local CDCs could undertake interventions to reduce the stigma of sex work, improve HIV/ADIS education and remove discriminatory perceptions as a barrier to HIV testing; moreover, priority should be given to expanding the participation of peer educators. Furthermore, as knowledge of HIV/AIDS and HIV testing increases, the extension of HIV testing services should be promoted, such as providing self-testing kit at pharmacies, making HIV testing as a routine service in all health posts, and promoting home-based testing, etc.

We recognize several limitations of our study. Firstly, due to the cross-sectional design of this study, we were unable to determine whether certain behaviors occurred before or after HIV testing, which limited our ability to establish causal relationship. Secondly, because the participants in our study were anonymous and the time-series samples in the same cities were used for three consecutive years, multiple presenting FSWs might result in sample overlap across years. Thirdly, as our survey involved some stigma-related behaviours, a social desirability bias may have resulted in under-reporting of high-risk behaviours. Despite these limitations, the current study represents one of the few large-scale studies to investigate HIV prevalence, uptake of HIV testing, and related factors among FSWs, including cross-border migrant Vietnamese FSWs, in the Sino-Vietnam border areas in China. In addition, this study has the advantage of examining a wide range of explanatory factors and using a large representative population sample, by which providing insights into HIV testing for FSWs in cross-border areas.

## Conclusions

Findings from this study have important implications for developing intervention programs targeting Sino-Vietnamese areas in China. First, because the rate of HIV testing uptake among Sino-Vietnam border FSWs in Guangxi, China was low, two aspects can be considered to improve the uptake of HIV testing: the accessibility of HIV testing provided by the government and the improvement of personal awareness. Second, since age, working condition, education level and location were the factors influencing uptake of HIV testing, future interventions should target those working in low-tier venues, older, less educated, and cross-border migrant Vietnamese FSWs. Third, since free AIDS education, free condom programs, peer education were the important factors promoting uptake of HIV testing, these approaches may be more effective in enhancing HIV testing in the future. Last, In addition to traditional intervention sites, STD clinics can also be convenient places to disseminate AIDS education materials.

## Supplementary Information

**Additional file 1:** Key population quantitative questionnaires.

## Data Availability

The datasets generated and/or analysed during the current study are not publicly available because of ethical and legal reasons but are available from the corresponding author Li Ye on reasonable request.

## Abbreviations

FSWs: Female sex workers
CL-FSWs: Chinese local FSWs
CMV-FSWs: Cross-border migrant Vietnamese FSWs
AIDS: Acquired immunodeficiency syndrome
HIV: Human immunodeficiency virus
STD: Sexual transmitted disease
STIs: Sexual transmitted infections
CDC: Centre for Disease Control and Prevention
UNAIDS: The Joint UnitedNations Programme on HIV and AIDS
WHO: The World Health Organization
VCT: Voluntary Counselling and Testing
MSM: Men who have sex with men
CAFTA: China-ASEAN free trade area
COR: Crude odds ratio
AOR: Adjusted odds ratio
CI: Confidence interval

## References

[1] Tucker JD, Wong FY, Nehl EJ, Zhang F (2012). HIV testing and care systems focused on sexually transmitted HIV in China. Sex Transm Infect.

[2] Zhao DH, Hui S, Song X, Tong X, Ma J, Zhang XL, Yuan LL, Yu Y (2022). Effects of unsafe sexual behavior and sexual orientation on previous HIV testing and HIV testing willingness among college students in Harbin. Zhonghua Liu Xing Bing Xue Za Zhi.

[3] Zhang X, Wang N, Vermund SH, Zou H, Li X, Zhang F, Qian HZ (2019). Interventions to improve the HIV continuum of care in China. Curr HIV/AIDS Rep.

[4] Understanding Fast-Track: accelerating action to end the AIDS epidemic by 2030. https://www.unaids.org/sites/default/files/media_asset/201506_JC2743_Understanding_FastTrack_en.pdf.

[5] Baral S, Beyrer C, Muessig K, Poteat T, Wirtz AL, Decker MR, Sherman SG, Kerrigan D (2012). Burden of HIV among female sex workers in low-income and middle-income countries: a systematic review and meta-analysis. Lancet Infect Dis.

[6] Wang J, Ding G, Zhu Z, Zhou C, Wang N (2015). Analysis of HIV correlated factors in Chinese and Vietnamese female sex workers in Hekou, Yunnan Province, a Chinese Border Region. PLoS ONE.

[7] Yu YJ, Li X, Tam CC, Zhou Y, Chen Y, Shen Z (2016). Demographic and behavioral correlates of HIV/STI among Vietnamese female sex workers in southwest China. AIDS Care.

[8] Yu J, Nehl EJ, Dinh VP, Liang B, Son NV, Meng D, Zhang Y, Jiang J, Huang J, Ning C (2020). Vietnamese female sex workers in rural cross-border areas of Guangxi, China: migration and HIV/STI risk behaviors. AIDS Care.

[9] Wang L, Tang W, Wang L, Qian S, Li YG, Xing J, Li D, Ding Z, Babu GR, Wang N (2014). The HIV, syphilis, and HCV epidemics among female sex workers in China: results from a serial cross-sectional study between 2008 and 2012. Clin Infect Dis.

[10] Chow EP, Muessig KE, Yuan L, Wang Y, Zhang X, Zhao R, Sun P, Sun X, Tucker JD, Jing J (2015). Risk behaviours among female sex workers in China: a systematic review and data synthesis. PLoS ONE.

[11] Lai J, Qin C, Nehl EJ, Jiang J, Huang Y, Liang B, Xu Y, Huang J, Xu Z, Ning C (2018). HIV prevalence among female sex workers in Guigang City, Guangxi, China: an 8-year consecutive cross-sectional study. BMC Public Health.

[12] Chen H, Luo L, Pan SW, Lan G, Zhu Q, Li J, Zhu J, Chen Y, Shen Z, Ge X (2019). HIV Epidemiology and Prevention in Southwestern China: Trends from 1996–2017. Curr HIV Res.

[13] Reilly KH, Wang J, Zhu Z, Li S, Yang T, Ding G, Qian HZ, Kissinger P, Wang N (2012). HIV and associated risk factors among male clients of female sex workers in a Chinese border region. Sex Transm Dis.

[14] Zhang H, Hsieh E, Wang L, Liao S (2020). HIV/AIDS among female sex workers in China: epidemiology and recent prevention strategies. Curr HIV/AIDS Rep.

[15] Hu X, Liang B, Zhou C, Jiang J, Huang J, Ning C, Liu J, Zhou B, Zang N, Lai J (2019). HIV late presentation and advanced HIV disease among patients with newly diagnosed HIV/AIDS in Southwestern China: a large-scale cross-sectional study. AIDS Res Ther.

[16] Hong Y, Zhang C, Li X, Fang X, Lin X, Zhou Y, Liu W (2012). HIV testing behaviors among female sex workers in Southwest China. AIDS Behav.

[17] Tokar A, Broerse JEW, Blanchard J, Roura M (2018). HIV testing and counseling among female sex workers: a systematic literature review. AIDS Behav.

[18] Wang Y, Pan JB, Wang XF, Li B, Henderson G, Emrick CB, Sengupta S, Cohen M (2010). Reported willingness and associated factors related to utilization of voluntary counseling and testing services by female sex workers in Shandong Province, China. Biomed Environ Sci.

[19] Li-li X, Tian Y (2020). The characteristics and development strategy of China's Land Border Ports. Guizhou Ethnic Studies.

[20] Zhang Y, Liang B, Liu D, Wei G, Mo S, Nong A, Ning C, Liao Y, Jiang J, Pan P (2020). Migrant female sex workers working at the Sino-Vietnamese border for a short time have a higher risk of HIV transmission: a consecutive cross-sectional study. AIDS Res Ther.

[21] Chen Y, Shen Z, Morano JP, Khoshnood K, Wu Z, Lan G, Zhu Q, Zhou Y, Tang S, Liu W (2015). Bridging the epidemic: a comprehensive analysis of prevalence and correlates of HIV, hepatitis C, and syphilis, and infection among female sex workers in Guangxi Province, China. PLoS ONE.

[22] The State Council AIDS Working Committee Office tPsRoC: China HIV/AIDS monitoring and evaluation framework (trial). Beijing: Beijing medical publishing house; 2007.

[23] Fan YG, Liu JJ, Zhang YJ, Dai SY, Li MQ, Ye DQ (2015). HIV, other sexually transmitted infections, and risk behaviors among female sex workers in Liuzhou, China. Int J Gynaecol Obstet.

[24] Wang W, Chen R, Ma Y, Sun X, Qin X, Hu Z (2018). The impact of social organizations on HIV/AIDS prevention knowledge among migrants in Hefei, China. Global Health.

[25] World Health Organization. In: Delivering HIV test results and messages for re-testing and counselling in adults. edn. Geneva, Switzerland. 2010. 26269878

[26] World Health Organization. In: Consolidated Guidelines on HIV Testing Services: 5Cs: Consent, Confidentiality, Counselling, Correct Results and Connection 2015. edn. Geneva, Switzerland; 2015. 26378328

[27] Annual Report of China National HIV/STD/HCV Comprehensive Prevention and Treatment Programs in 2018. Beijing: National Center for AIDS/STD Control and Prevention, China CDC; 2019.

[28] Zhang H, Hsieh E, Wang L, Liao S (2020). HIV/AIDS among female sex workers in China: epidemiology and recent prevention strategies. Curr HIV/AIDS Rep.

[29] Hao C, Liu H, Sherman SG, Jiang B, Li X, Xu Y, Jiang Z, Zang C (2014). Typology of older female sex workers and sexual risk for HIV infection in China: a qualitative study. Cult Health Sex.

[30] Xu J, Brown K, Ding G, Wang H, Zhang G, Reilly K, Li Q, Wang G, Wang N (2011). Factors associated with HIV testing history and HIV-test result follow-up among female sex workers in two cities in Yunnan, China. Sex Transm Dis.

[31] Shokoohi M, Noori A, Karamouzian M, Sharifi H, Khajehkazemi R, Fahimfar N, Hosseini-Hooshyar S, Kazerooni PA, Mirzazadeh A (2017). Remaining gap in HIV testing uptake among female sex workers in Iran. AIDS Behav.

[32] King EJ, Maman S, Dudina VI, Moracco KE, Bowling JM (2017). Motivators and barriers to HIV testing among street-based female sex workers in St. Petersburg, Russia. Glob Public Health.

[33] Tokar A, Sazonova I, Mishra S, Smyrnov P, Saliuk T, Lazarus JV, Broerse JEW, Roura M, Blanchard J, Becker ML (2019). HIV testing behaviour and HIV prevalence among female sex workers in Ukraine: findings from an Integrated Bio-Behavioural Survey, 2013–2014. Sex Transm Infect.

[34] Tran BX, Nguyen LT, Nguyen NP, Phan HT (2013). HIV voluntary testing and perceived risk among female sex workers in the Mekong Delta region of Vietnam. Glob Health Action.

[35] Wang B, Li X, Stanton B, McGuire J (2010). Correlates of HIV/STD testing and willingness to test among rural-to-urban migrants in China. AIDS Behav.

[36] Wang C, Wang Y, Tucker J, Xiong M, Fu H, Smith M, Tang W, Ong J, Zheng H, Yang B (2020). Correlates of HIV self-testing among female sex workers in China: implications for expanding HIV screening. Infect Dis Poverty.

[37] Wang Y, Li B, Pan J, Sengupta S, Emrick CB, Cohen MS, Henderson GE (2011). Factors associated with utilization of a free HIV VCT clinic by female sex workers in Jinan City, Northern China. AIDS Behav.

[38] Lau CYK, Wang Z, Fang Y, Ip M, Wong KM, Chidgey A, Li J, Lau JTF (2021). Prevalence of and factors associated with behavioral intention to take up home-based HIV self-testing among male clients of female sex workers in China—an application of the Theory of Planned Behavior. AIDS Care.

[39] Fang Y, Zhang Y, Wang Z, Ip M, Li J, Lau JTF (2018). Low uptake of HIV testing among male clients of female sex workers in China. AIDS Care.

[40] Teklehaimanot HD, Teklehaimanot A, Yohannes M, Biratu D (2016). Factors influencing the uptake of voluntary HIV counseling and testing in rural Ethiopia: a cross sectional study. BMC Public Health.

