# Correction to: Trends and associated factors in the uptake of HIV testing among female sex workers in Sino-Vietnam border areas in Guangxi, China: a cross-sectional study

**DOI:** 10.1186/s12879-022-07521-0

**Published:** 2022-06-15

**Authors:** Bingyu Liang, Qianni Huang, Yanyun Ou, Fei Zhang, Peidong Zhang, Aidan Nong, Shide Mo, Zhenxian Wu, Hai Xie, Huayue Liang, Jie Liu, Junjun Jiang, Hao Liang, Deping Liu, Li Ye

**Affiliations:** 1 Guangxi Key Laboratory of AIDS Prevention and Treatment, School of Public Health, Guangxi Medical University, Nanning, 530021 Guangxi China; 2 Guangxi Collaborative Innovation Center for Biomedicine, Life Sciences Institute, Guangxi Medical University, Nanning, 530021 Guangxi China; 3 Chongzuo Center for Disease Control and Prevention, Chongzuo, 532200 Guangxi China; 4 Fangchenggang Center for Disease Control and Prevention, Fangchenggang, 538000 Guangxi China

## Correction to: BMC Infectious Diseases (2022) 22:479 10.1186/s12879-022-07459-3

Following publication of the original article [1], the authors identified some errors in Tables 2 and 3. The correct tables (Tables 2, 3) are given in this correction.

**Table 2:** Univariate and multivariate logistic regression of socio-demographic factors for HIV testing in the preceding year among Sino-Vietnam border female sex workers, 2016–2018 (n = 5201)

Variables	Total tested	Cross-border migrant Vietnamese FSWs					Chinese local FSWs				
		Tested	COR (95% CI)	*p*	AOR (95% CI)	*p*	Tested	COR (95% CI)	*p*	AOR (95% CI)	*p*
Age											
≥ 35 years old	1230 (44.23)	164 (39.71)	1		1		1066 (42.78)	1		1	
< 35 years old	1551 (55.77)	125 (56.05)	1.94 (1.39–2.69)	< 0.001	1.93 (1.20–3.10)	0.01	1426 (68.79)	2.95 (2.61–3.33)	< 0.001	1.89 (1.60–2.24)	< 0.001
Ethnicity											
Han	958 (34.45)	–	–	–	–	–	958 (53.94)	0.85 (0.68–1.06)	0.15	–	–
Zhuang	1307 (47.00)	–	–	–	–	–	1305 (54.51)	0.87 (0.70–1.08)	0.20	–	–
Other	516 (18.55)	–	–	–	–	–	229 (57.97)	1			
Marital status											
Married, cohabiting	2069 (74.40)	217 (50.35)	1.87 (1.33–2.64)	< 0.001	1.34 (0.84–2.13)	0.22	1852 (59.88)	1.94 (1.71–2.20)	< 0.001	0.94 (0.80–1.11)	0.46
Single	712 (25.60)	72 (35.12)	1		1		640 (43.48)	1		1	
Education											
≥ 9 years	1705 (61.30)	209 (78.28)	3.05 (2.19–4.26)	< 0.001	1.75 (1.09–2.82)	0.02	1496 (88.89)	0.74 (0.66–0.84)	< 0.001	1.13 (0.96–1.33)	0.13
< 9 years	1076 (38.70)	80 (21.68)	1		1		996 (34.56)	1		1	
Location											
Chongzuo	2358 (84.79)	238 (61.66)	6.28 (4.34–9.08)	< 0.001	3.23 (1.70–6.11)	< 0.001	2120 (65.01)	4.66 (4.05–5.36)	< 0.001	3.67 (3.07–4.38)	< 0.001
Fangchenggang	423 (15.21)	59 (0.24)	1		1		372 (28.53)	1		1	

*COR* crude odds ratio, *AOR* adjusted odds ratio, *CI* confidence interval

**Table 3:** Univariate and multivariate logistic regression of behavioral factors and psychological factors for HIV testing in the preceding year among Sino-Vietnam border female sex workers, 2016–2018 (n = 5201)

Variables	Total tested	Cross-border migrant Vietnamese FSWs					Chinese local FSWs				
		Tested	COR (95% CI)	*P*	AOR (95% CI)	*P*	Tested	COR (95% CI)	*P*	AOR (95% CI)	*P*
Having adequate HIV-/AIDS-related knowledge											
Yes	2681 (96.40)	269 (47.36)	2.16 (1.25–3.73)	0.01	0.85 (0.41–1.80)	0.68	2412 (54.71)	1.15 (0.83–1.58)	0.40	–	–
No	100 (3.60)	20 (29.41)	1		1		80 (51.28)	1		–	–
Working venue type											
High-/Mid-tier	1425 (51.24)	138 (66.99)	3.75 (2.64–5.32)	< 0.001	5.11 (2.83–9.21)	< 0.001	1287 (50.47)	0.68 (0.61–0.77)	< 0.001	1.0 (0.84–1.20)	0.96
Low-tier	1356 (48.76)	151 (35.12)	1		1		1205 (50.80)	1		1	
Age of sexual debut											
< 18 years	442 (15.89)	94 (51.37)	1.40 (0.99–1.97)	0.06	0.69 (0.43–1.09)	0.11	348 (52.97)	0.93 (0.79–1.10)	0.37	–	–
≥ 18 years	2339 (84.11)	195 (43.05)	1		1		2144 (54.86)	1		–	–
Age of commercial sexual debut											
≥ 18 years	2750 (98.89)	277 (45.19)	1		–	–	2473 (55.07)	1		–	–
< 18 years	31 (1.11)	12 (52.17)	1.32 (0.58–3.05)	0.51	–	–	19 (25.68)	0.28 (0.17–0.48)	< 0.001	–	–
Months of staying in the current residence											
≥ 12 months	1626 (58.47)	95 (42.60)	0.66 (0.47–0.93)	0.02	1.1 (0.68–1.83)	0.68	1531 (65.60)	2.06 (1.80–3.67)	< 0.001	1.28 (1.08–1.52)	0.04
6–11 months	322 (11.58)	16 (20.78)	0.23 (0.13–0.42)	< 0.001	0.56 (0.27–1.18)	0.13	306 (35.33)	0.59 (0.50–0.71)	< 0.001	0.58 (0.47–0.71)	< 0.001
< 6 months	830 (29.85)	178 (52.98)	1		1		652 (48.01)	1		1	
Years of FSW experience											
≥ 4 years	1450 (52.14)	79 (43.65)	0.90 (0.64–1.28)	0.57	–	–	1371 (57.92)	1.32 (1.18–1.49)	< 0.001	1.61 (1.38–1.88)	< 0.001
< 4 years	1331 (47.86)	210 (46.15)	1		–	–	1121 (51.00)	1		1	
Consistent condom used in last commercial sex											
Yes	2742 (98.60)	280 (45.45)	1.02 (0.42–2.49)	0.97	–	–	2462 (55.09)	1.61 (0.99–2.62)	0.06	1.55 (0.83–2.87)	0.17
No	38 (1.40)	9 (45.00)	1		–	–	29 (43.28)	1		1	
Consistent condom used in the previous month											
Yes	2540 (91.33)	236 (43.30)	1		1		2304 (54.21)	1		1	
No	241 (8.67)	53 (58.24)	1.83 (1.17–2.86)	0.01	1.94 (1.07–3.53)	0.03	188 (59.68)	1.25 (0.10–1.58)	0.06	1.56 (1.15–2.11)	0.004
History of self-reported STIs											
Yes	154 (5.54)	14 (73.68)	3.48 (1.24–9.79)	0.02	4.25 (1.31–13.76)	0.02	140 (61.14)	1.33 (1.01–1.74)	< 0.001	1.07 (0.79–1.46)	0.79
No	2627 (94.46)	275 (44.57)	1		1		2352 (54.24)	1		1	
Ilicit drug use											
Yes	52 (1.87)	3 (50.00)	1.20 (0.24–6.01)	0.82	–	–	49 (64.47)	1.52 (0.95–2.44)	0.08	–	–
No	2729 (98.13)	286 (45.40)	1		–	–	2443 (54.42)	1		–	–
Serving clients in a fixed venue											
Yes	2514 (90.40)	281 (46.68)	2.84 (1.27–6.39)	0.01	1.08 (0.39–3.04)	0.88	2233 (56.23)	1.66 (1.40–2.00)	< 0.001	1.44 (1.17–1.79)	0.001
No	267 (9.60)	8 (23.53)	1		1		259 (43.60)	1		1	
Having received free AIDS education and condom distribution services in the past year											
Yes	2762 (99.32)	284 (47.33)	5.57 (2.14–14.53)	< 0.001	1.77 (0.58–5.39)	0.32	2478 (55.37)	6.74 (3.80–11.95)	< 0.001	2.93 (1.92–2.67)	< 0.001
No	19 (15.18)	5 (13.89)	1		1		14 (15.56)	1		1	
Having received peer education services in the past year											
Yes	1820 (65.44)	243 (59.27)	5.69 (3.90–8.32)	< 0.001	1.1 (0.59–2.08)	0.76	1577 (69.47)	3.43 (3.04–3.88)	< 0.001	2.26 (1.92–2.66)	< 0.001
No	961 (34.56)	46 (20.35)	1		1		915 (39.86)	1		1	
Number of clients in the last month											
< 60	1772 (63.72)	192 (48.73)	1		1		1580 (57.69)	1		1	
≥ 60	1009 (36.28)	97 (40.08)	0.70 (0.51–0.97)	0.03	1.30 (0.85–1.98)	0.24	912 (49.95)	0.73 (0.65–0.82)	< 0.001	1.66 (1.41–1.94)	< 0.001
Average age of male clients											
≥ 50 years	820 (29.49)	68 (48.23)	1.16 (0.79–1.68)	0.45	–	–	752 (69.05)	2.23 (1.93–2.57)	< 0.001	1.03 (0.85–1.25)	0.76
< 50 years	1961 (70.51)	221 (44.65)	1		–	–	1740 (50.06)	1		1	
Having regular, non-commercial sexual partners											
Yes	2329 (83.75)	15 (44.12)	0.94 (0.47–1.90)	0.87	–	–	437 (71.75)	2.35 (1.95–2.83)	< 0.001	2.10 (1.63–2.62)	< 0.001
No	452 (16.25)	274 (45.51)	1		–	–	2055 (51.95)	1		1	
Having regular, commercial sexual partners											
Yes	1737 (62.46)	205 (50.74)	1.80 (1.30–2.53)	< 0.001	1.59 (1.03–2.46)	0.04	1532 (60.82)	1.76 (1.56–1.98)	< 0.001	1.46 (1.25–1.70)	< 0.001
No	1044 (37.54)	84 (36.21)	1		1	–	960 (46.92)	1		–	–
Clients' average payment for commercial sex											
< 100 RMB	1174 (42.22)	196 (52.83)	2.07 (1.50–2.87)	< 0.001	2.40 (1.36–3.68)	0.001	978 (59.56)	1.37 (1.21–1.55)	< 0.001	0.81 (0.67–0.97)	0.02
≥ 100 RMB	1607 (57.78)	93 (35.09)	1		1		1514 (51.80)	1		1	
Perception of risk for HIV infection											
High level	363 (13.05)	41 (50.00)	1		1		322 (49.31)	1			
Mid level	2091 (75.19)	238 (47.22)	0.89 (0.56–1.43)	0.64	1.00 (0.58–1.73)	1	1853 (56.67)	1.34 (1.14–1.59)	0.001	–	–
Low level	327 (11.76)	10 (20.00)	0.25 (0.11–0.57)	0.001	0.98 (0.36–2.67)	0.97	317 (49.38)	1.10 (0.80–1.25)	0.98	–	–
Perception of vulnerability to HIV/STIs											
Yes	2220 (79.83)	276 (50.00)	5.46 (2.96–10.10)	< 0.001	2.43 (1.17–5.05)	0.02	1944 (59.56)	2.02 (1.78–2.30)	< 0.001	1.72 (1.45–2.03)	< 0.001
No	561 (20.17)	13 (15.48)	1		1		548 (42.12)	1		1	
Fearing their families knowing about their sex work											
Yes	1880 (67.60)	198 (56.09)	2.69 (1.95–3.74)	< 0.001	0.81 (0.50–1.33)	0.41	1682 (62.25)	2.10 (1.90–2.42)	< 0.001	1.56 (1.34–1.81)	< 0.001
No	901 (32.40)	91 (32.16)	1		1		810 (43.48)	1		1	

*COR* crude odds ratio, *AOR* adjusted odds ratio, *CI* confidence interval

The original article [1] has been corrected.
