# Supplementary material for: Trends and associated factors in the uptake of HIV testing among female sex workers in Sino-Vietnam border areas in Guangxi, China: a cross-sectional study
Source: BMC Infect Dis. 2022 May 19;22:479. doi: 10.1186/s12879-022-07459-3 (PMC9118634; doi:10.1186/s12879-022-07459-3)
Supplement: Supplementary file 1 — Additional file 1: Key population quantitative questionnaires. [file 12879_2022_7459_MOESM1_ESM.pdf]

Key population quantitative questionnaires

Introduction:

Hello, We are conducting a survey mainly to investigate your awareness of health knowledge, attitude and behavior. It may involve some of your privacy, but the content of your answers will be kept strictly confidential. We only use these questionnaires to do population analyses, not to do personal analyses. We won't disclose your information to any other individuals or organizations. So we hope you answer the following questions truthfully. This investigation will take you some time. We have to say sorry for any convenience caused. Thank you for your support.

Questionnaire based information

No.	**Questions and options**
A01	**Survey location:** ___________ province (municipality) ___________city/district/county
A02	**Informed consent:** _______________ **(Yes/No)**
A03	**Questionnaire number** □□□□□□□□□□ (administrative division GB code + investigation object category + investigation object number)
A04	**Number of cohort surveys** ①Baseline ②First follow-up
A05	**Investigation date** date:___________
A06	**Working Venue** ①sauna/bath center ②nightclub ③Karaoke bar/ dance halls/ pub-bar ④high-cost hotel ⑤ mid-ranking hotels ⑥rental house/client's house ⑦foot bath salon/hair salon ⑧shop/small restaurant ⑨ street/roadside/park ⑩others (please note: _____________)
A07	**The investigation site：** ①Urban area (county or city) ②Urban-rural fringe ③ villages and towns ④village

Socio-demographic, behavioural and psychological questions

No.	Questions and options	Answers
B01	**Your birthday?** ____ year____month_____day	
B02	**Your marital status?** ① unmarried ② married ③ cohabit ④ divorced or widowed	
B03	**You registered residence?** ① local province<br>②outside Guangxi province(please note: _____________)<br>③ other country（please note:______________ country）	
B04	**Your nationality?**	
B05	**What is your degree of education?**<br>① Illiteracy or semi-literate ② Primary school ③ Middle school ④ High school/ technical secondary school/ vocational high school<br>⑤ Junior college and above ⑥Graduate degree and above	
B06	**How long have you ever been working in this county or city ?**<br>① >=12 months ② 6 -11 months ③1-6 months ④ 1<months	
B07	**Where were your last working place ?**<br>① Outside province ② Local province but outside this city ③Local city ④ None	
B08	**How many months have you been doing this work (commercial sexual service) in total?**	__________month(s)
B09	**How many months do you usually do this work in a city (or county) ?**	__________month(s)

**HIV/AIDS knowledge**		
C01	**Is AIDS a serious infectious disease with no cure?** ①Yes ②No ③ I don't know	
C02	**Is sexual transmission the main mode of HIV transmission in China?**<br>①Yes ②No ③ I don't know	
C03	**Can you tell if someone is infected HIV from the appearance of their genitals?** ①Yes ②No ③ I don't know	
C04	**Does it increase the risk of HIV infection if someone has a sexually transmitted disease?** ①Yes ②No ③ I don't know	
C05	**Can condom use reduce the risk of infecting and transmitting HIV?** ①Yes ②No ③ I don't know	
C06	**Does new type of drug use (such as methamphetamine, ecstasy, ketamine, etc.) increase the risk of HIV infection?**<br>①Yes ②No ③ I don't know	
C07	**Should someone actively seek HIV testing and counseling after high risk behaviors (needle sharing, drug use, unsafe sex, etc.)?**<br>①Yes ②No ③ I don't know	
C8	**Is there legal liability for intentionally spreading HIV?** ①Yes ②No ③ I don't know	
**Sexual behaviour characteristics**		
D01	**How old were you when you first started sexual life ?**	_________years old
D02	**How old were you when you first engaged in commercial sex ?**	_________years old
D03	**Did your clients use condom in the last commercial sex ?** ①Yes ②No	

D04	**Last a month, how often do your clients use condoms ?** ①Never ②Sometimes ③Every time	
D05	**How many clients did you serve on the last working day ?**	_____individual(s)
D05-01	**How many regular commercial sexual partners among them ?**	_____individual(s)
D06	**How many clients did you serve last week ?**	_____individual(s)
D06-01	**How many regular commercial sexual partners among them ?**	_____individual(s)
D07	**How many clients did you serve last month ?**	_____individual(s)
D07-01	**How many regular commercial sexual partners among them ?**	_____individual(s)
D08	**How much do you charge for a commercial sex in average ?**	_____RMB(Yuan)
D09	**How much do you charge for a commercial sexual behavior without condom?**（If never, please note “No” ）	①_____RMB(Yuan)<br>② No
D10	**How much do you charge for a commercial sex with condom?**	_____RMB(Yuan)
D11	**How much is your annual income working as a female sex worker ?**	_____RMB(Yuan)
D12	**In the past six month, how did you look for clients?** （multiple choices）<br>① In a fixed venues (including saunas place, nightclubs, bars, hotels, hair salons, roadside shops, etc.)<br>② Telephone appointment or door-to-door service ③ Internet (including QQ, Wechat, Momo, website,etc.)	Choices:_________,<br>Main choices:____<br>（simple choice）
D13	**What’s the average age of your male clients?**<br>① under 29 ②30-49 years old ③over 50 year old	

D14	**Whether your clients have taken aphrodisiacs before you served them ?**<br>①No (Skip to D17）②sometimes ③Usually	
D15	**What are the age of the clients who use aphrodisiacs (or use other aphrodisiac tool)?（multiple choices）**<br>①Under 40 ②40-50 years old ③50-60 years old ④60-70 years ⑤more than 70 years old ⑥unknown	
D16	**Did you think it was effective after they use aphrodisiacs?** ①No ②Yes ③Unknown	
D17	**Do you have a regular, non-commercial sexual partners?** ①Yes, _______individual(s) ② No (Skip to E01)	
D18-01	**Where are your non-commercial sexual partners come from?______**(County or viliage)	
D18-02	**How many times does he look for you in a month?**<br>① less than once a month ② once or twice a month ③ 3-5 times a month<br>④ more than 5 but less than 9 times a month ⑤ more than 10 times a month	
**Following questions only ask if participant was married or cohabiting with a boyfriend**		
E01	**How often have you sex with your spouse or boyfriend ?** ①more than one time a week ② less than one time a week ③never	
E02	**How often do your spouse or boyfriend use condom when you have sex with him ?**①never ② sometime ③every time	
**HIV-risk behaviour**		
F01	**Do you use any illicit drug? Or have you ever used illicit drug ? (Including heroin, cocaine, opium, marijuana, morphine, methamphetamine, doloretine, K powder (ketamine), ecstasy, and cannabis)** ①Yes ②No	
F02	**Do you inject illicit drug? Or have you ever injected illicit drug ?** ①Yes ②No	
F03	**How many times did you inject illicit drug a day in average ?** _______________times	

F04	**Did you share needles with others when you inject illicit drug ?** ①Yes ②No	
F05	**How often did you share needles with others when you inject illicit drug in the past six months?**<br>①Never ②Sometime ③Every time	
**Psychological variables**		
G01	**Do you perceive the risk of HIV infection as a female sex worker ?**<br>①very likely ②likely ③unlikely ④very unlikely	
G02	**Do you perceive the vulnerability of HIV/STI infection as a female sex worker ?**<br>①very likely ②likely ③unlikely ④very unlikely	
G03	**What are you fear of as a female sex worker ? (multiple optional)**<br>① infected HIV ②infected STIs ③ Clients don't give money<br>④ Police arrest ⑤Families or acquaintances know my work ⑥ Others	
**HIV testing (self-reported)**		
H01	**Have you been diagnosed with a sexually transmitted disease in the past year?** ①Yes ②No (Skip to H03)	
H02	**What kinds of STDs have you been diagnosed with in the past year? (Multiple options)**<br>①gonorrhea ②syphilis ③Genital tract Chlamydia trachomatis infection ④condyloma acuminatum<br>⑤genital herpes ⑥Others____________	
H03	**Have you ever taken a HIV test (blood donations not included) in the preceding year ?** ①Yes ②No (Skip to J01)	
H04	**What's the date of the HIV test in the preceding year ?** ________year___________month___________day	
H05	**Where did you take the HIV test in the preceding year ?** ①Local area ②Other places_________	

H06	**What's the result of the HIV test in the preceding year ?** ①Negative ②Positive ③ I don't know	
**HIV-related services**		
I01	**Have you ever received free AIDS education and condom distribution services in the past year ?** ①Yes ②No	
I02	**Have you ever received free illicit drug maintenance therapy or clean needles provided by community or the local CDC in the past year ?** ①Yes ②No	
I03	**Have you ever received peer education services from the local CDC in the past year ?** ①Yes ②No	
**This the end of the investigation. Thank you for your cooperation. To understand your sexual health, we would like to collect your 5ml blood for HIV, syphilis and hepatitis C test. The test is free for you, and we will tell you the result of the test.**		
J01	**Would you like to donate blood and be tested for sexual transmitted disease after the investigation ?** ①Yes ②No	
J02a	**Have you ever tested positive for HIV before ?** ①Yes ②No (Skip to J03)	
J02b	**If yes, what was the earliest date of the positive test ?**	Date: _______________
**The following questions should be filled out by the investigator based on the test results.**		
J03	**HIV antibody test results**	
	**First ELISA screening** ①Positive ②Negative	
	**Second ELISA recheck** ①Positive ②Negative	
	**Confirmed test** ①Positive ②Negative ③Suspicious ④No detected	
J04	**Syphilis test result**	

	**ELISA screening** ①Positive ②Negative	
	**RPR/TRUST detection** ①Positive ②Negative	
J05	**HCV test result**	
	**First ELISA screening** ①Positive ②Negative	
	**Second ELISA re-test** ①Positive ②Negative	

The questionnaire quality control table

Item	Completion situation		Note
	Yes	No	
Specification, clear			
All questions were answered, no leakage			
No logical errors			

Note: Please put "√" in accord with the options

Investigator sign: ____________ Investigation date: __________________

Quality control supervisor sign: ____________ Date: _________________
